# Two-Photon Laser Scanning Stereomicroscopy for Fast Volumetric Imaging

**DOI:** 10.1371/journal.pone.0168885

**Published:** 2016-12-20

**Authors:** Yanlong Yang, Baoli Yao, Ming Lei, Dan Dan, Runze Li, Mark Van Horn, Xun Chen, Yang Li, Tong Ye

**Affiliations:** 1 State Key Laboratory of Transient Optics and Photonics, Xi’an Institute of Optics and Precision Mechanics, Chinese Academy of Sciences, Xi’an, China; 2 Department of Radiology, Medical University of South Carolina, Charleston, South Carolina, United States of America; 3 Department of Bioengineering and The Center for Optical Materials Science and Engineering Technologies (COMSET), Clemson University, Clemson, South Carolina, United States of America; 4 Department of Regenerative Medicine and Cell Biology, Medical University of South Carolina, Charleston, South Carolina, United States of America; Pennsylvania State Hershey College of Medicine, UNITED STATES

## Abstract

Bessel beams have been successfully used in two-photon laser scanning fluorescence microscopy to extend the depth of field (EDF), which makes it possible to observe fast events volumetrically. However, the depth information is lost due to integration of fluorescence signals along the propagation direction. We describe the design and implementation of two-photon lasers scanning stereomicroscopy, which allows viewing dynamic processes in three-dimensional (3D) space stereoscopically in real-time with shutter glasses at the speed of 1.4 volumes per second. The depth information can be appreciated by human visual system or be recovered with correspondence algorithms for some cases.

## Introduction

Revealing the dynamics of physiological processes in their native environment with cellular or subcellular resolution is currently a major goal in optical microscopy. The capability of imaging in three dimensions, i.e., volumetric imaging, is essential because of the 3D nature of physiological processes. The two fundamental ways to acquire volumetric images are tomographic imaging and localized imaging [1]. Tomographic imaging detects line-projection signals from multiple viewing angles and reconstructs 3D images by algorithms based mainly on the projection-slice theorem [2]. Medical imaging methods, such as X-ray computed tomography (CT) and magnetic resonant imaging (MRI), are typical tomographic imaging methods that provide 3D images at the organ level. The localized imaging establishes a one-to-one relation between each voxel and its corresponding small volume (focal volume) in a specimen and reconstructs 3D images by scanning all points, throughout the imaged volume. Since the focal volume can be made as small as femtoliters [3], localized imaging is suitable for high-resolution microscopy at the cellular or subcellular level. Localized imaging is the basis of most of laser scanning microscopy, such as confocal microscopy [4] and two-photon fluorescence microscopy (TPM) [5]. However, because these methods acquire each voxel of a volumetric image sequentially, meeting the required imaging speed for physiological studies is difficult.

The imaging speed can be improved by using one of the following approaches [6]: increasing scanning speed, increasing the number of pixels collected in parallel or selecting a small set of pixels to acquire. Many types of scanning hardware, such as the resonant scan mirror, polygon scanner and acousto-optic deflector, have been employed to increase scanning speed. The recently reported optical phase-locked ultrasound lens [7] (OPLUL) has achieved the fastest scanning speed to date in TPM. However, as pixel dwell time decreases, the number of detectable photons per pixel decreases as does the signal-to-noise ratio. Scanning multiple focal points created by diffractive optical element or lenslet array [8–10] or using selective plane illumination [11] can increase the parallelism of excitation. These schemes also have excellent optical sectioning capability either by spinning-disk confocal configuration [10] or by orthogonal detection [12–14]. However, these schemes need a detector array (multianode photomultiplier tube [9]) or a camera (CCD or SCMOS) [10–14] for wide-field imaging. They are suitable for imaging weak scattered or transparent specimens but suffer from light scattering when imaging the turbid tissue [15]. A large area single pixel detector, such as photomultiplier tube (PMT) is superior for collecting scattered photons in sequential scanning approach. Random access scanning is another method that can acquire a prior defined set of points in the volume at up to hundreds of Hertz [16]. The requirement of prior knowledge makes random access scanning unsuitable for imaging moving objects.

**Fig 1:**
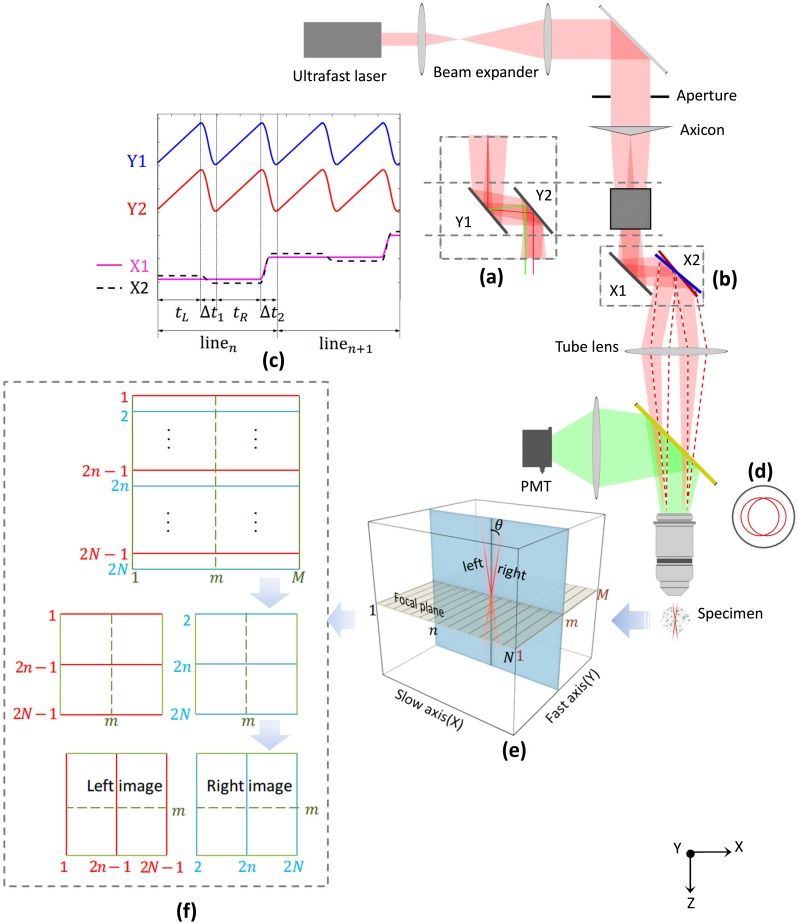
The principle of TPLSSM. (a) Side view of the Y scanning pair. The red and green lines show two parallel scanning positions. (b) The X scanning pair. X2 serves as an angle switcher for tilting the focus. (c) Control waveforms of the Stereo-scanner. (d) Switchable annular illumination at BFP. (e) Stereoscopically scanning with tilted Bessel beams. Scanning geometry with N×M scanning positions with each position excited twice from different angles (f) Data processing of interlaced raw images. From top row to bottom row: Interlaced raw data with odd and even lines for left (red) and right (cyan) views, respectively; Deinterlaced into two matrices; Rotated 90° and to create a stereo-pair. The acquired stereo-pair can be directly viewed with 3D glasses or processed by correspondence algorithm for depth recovering.

Here we report on two-photon laser scanning stereomicroscopy (TPLSSM, Fig 1), a new approach that can achieve fast volumetric imaging based on a conventional two-photon fluorescence microscope. Herein, we demonstrate that volume images of specimens can be acquired and displayed stereoscopically on a 3D monitor in real-time. The depth information can be recovered by using stereo correspondence algorithms. Since only two scanned images from different angles are required, volumetric imaging speed is greatly improved.

## Methods

### Stereo-scanner for Bessel beams and system setup

The three essential requirements for constructing a stereomicroscope are the extended depth of field (EDF, see [17] for review) to make a large volume of the sample in focus; two viewing angles to form parallax; and an appropriate simultaneity of imaging the two views to co-register the features to enable a successful depth perception. Commonly used in conventional two-photon microscopy, the focused Gaussian beam has a very limited focusing depth and cannot provide the necessary extension along the propagation direction. Fortunately, the Bessel beam [18] (experimentally obtained as Bessel-Gauss beam) can form an extended focus that would be suitable for scanning a volume. Botcherby et al. [19] demonstrated that tilted Bessel beams could be realized to generate the parallax that stereomicroscopy needed. Following the same idea, Thériault et al. [20] and we [21] demonstrated that high-resolution stereoscopic images could be acquired by scanning Bessel beams with conventional 2D scanning module, with the parallax generated by manually shifting either the axicon or the tube lens laterally by using a mechanical stage. However, besides the cumbersome mechanical movement for creating parallax, such a stereoscopic imaging approach with successive frames for left and right views results in a time delay of seconds to minutes between different views. This destroys the simultaneity condition of stereoscopic imaging and, for some dynamic specimen, makes the contents of the captured stereo-pairs irrelevant.

To solve aforementioned problem, it is beneficial to generate parallax by tilting Bessel beams with a galvo mirror because of its fast response time. For this purpose, one needs to add one more degree of freedom of scanning to the conventional laser scanning system. This design would require three galvo mirrors, two of which are conjugated to the back focal plane (BFP) of objective lens to scan Bessel beams laterally, while the third one is conjugated to the focal plane for tilting the Bessel beams. For compactness, we adopted here a four-galvo configuration, previously used in STED microscopy [22] for beam stabilization purpose, to scan the Bessel beams at different tilted angles in TPLSSM. The Stereo-scanner, as termed here, consists of two galvo pairs (6210H, Cambridge Technology, MA) that correspond to X and Y scanning, respectively. Each galvo pair has two scanning mirrors rotating synchronously and being kept in parallel during scanning. This keeps the scanned Bessel beam always in parallel with the optical axis during scanning without introducing any aberration. The resultant lateral scanning pattern is relayed to the focal region by a tube lens and an objective. Tilting of the beam is realized by applying a tiny angle shift to make one scanning mirror in the slow galvo pair (X2 in Fig 1) off its parallel position. This angle causes a small displacement of the annular beam at the BFP of objective. Since the response time of the galvo mirror is about a few hundred microseconds for small angles, the angle switching is made very fast and almost unnoticeable. The Stereo-scanner can replace the conventional XY galvo scanner in the two-photon microscope, and delivers fast, stable and flexible scanning behavior that the TPLSSM would need.

Another remarkable feature of Stereo-scanner is the time delay between left and right views can be reduced to millisecond level by switching views line by line instead of frame by frame, as will be discussed below. Fig 1 shows a schematic diagram of the two-photon laser scanning stereomicroscopy system. An ultrafast Ti:Sapphire laser (MaiTai HP, Newport, CA) was tuned to 780nm for excitation. The beam was first expanded and then passed a customized axicon (base angle ≈1.15°), which converted the Gaussian beam to a Bessel-Gauss beam. The Y-axis galvo pair, shown in Fig 1a, then scanned the beam along the fast axis. Each line was scanned twice with differently tilted Bessel beams, causing stereoscopic excitation. In our system, X2 was the mirror used to switch the tilting angle by alternating its position between the red and blue line Fig 1b. The waveforms that drive the four mirrors are shown in Fig 1c. A stereo line pair is acquired during *t*_*L*_ and *t*_*R*_, respectively. The switch of the viewing angle happens during the flyback time Δ*t*_1_ and Δ*t*_2_. By carefully tuning the voltage waveforms of each galvo pair, two stable, annular beams with sharp focus were achieved at the BFP, and no jitter was observed during angle switching. The galvo control waveforms were generated by analog output channels of two DAQ boards (PCI-6110 and PCI-6713, National Instruments, TX), which were synchronized to each other with an RTSI bus cable. The emitted fluorescence was collected by a high NA objective lens (XLUMPLFLN 20X, 1.0 NA, Olympus, Japan) and detected by a PMT (PMM02, Thorlabs).

### Software control

**Fig 2:**
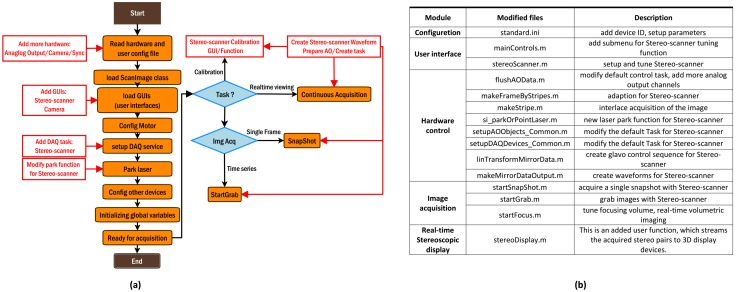
Software control framework based on ScanImage. (a) Modules modified (in red boxes) based on ScanImage workflow (from “Start” to “End”, see [22] for details). (b). List of all the modified files of ScanImage.

Stereoscopic image acquisition was controlled by ScanImage r3.8 [23], a widely used open-source software package written in Matlab (The MathWorks Inc., MA, USA) for home-built two-photon laser scanning microscopes. In order to accommodate it to TPLSSM, we modified or replaced part of the source code to implement the control of the Stereo-scanner and related stereo image acquisition. For example, we replaced the source code of waveform generation for providing the required voltage waveforms shown in Fig 1c to drive the Stereo-scanner. We also added a calibration module for Stereo-scanner to fine-tune the parameters of individual galvos and to adjust the parallax angle. A summary of all the modification is listed in Fig 2. The source code including all the modification to ScanImage and real-time stereoscopic display is available upon request.

### Real-time stereoscopic display

Stereoscopic techniques are commonly used for data visualization with the input of a 3D image stack projected along two different directions. Since the acquisition of a 3D image stack usually takes over tens of seconds in conventional TPM, it is impractical to stereoscopically observe the specimen in real-time. As TPLSSM acquires a stereo-pair that represent projections of a fluorescence volumetric image along two directions, it is possible to stream the captured stereo-pair on 3D display devices for real-time observation with light image processing.

According to the imaging process of TPLSSM, a raw image frame contains both left and right views with interlaced odd and even lines, respectively. A stereo-pair is created by separating the odd and even lines into two individual images, as shown in Fig 1f. The stereo-pair is displayed on a 3D-ready monitor using the stereo display module of Psychtoolbox [24], which is invoked through user defined functions of ScanImage. Wearing 3D glasses (NVIDIA 3D Vision Kit) with a compatible monitor (refresh rate > 120Hz) allows for real-time observation of specimens in 3D. The advantage of using 3D shutter glasses is that multicolor stereoscopic imaging is straightforward when incorporated with a multichannel detection system.

In general, this laser scanning stereomicroscopy performs volumetric imaging at a speed that is approximately half of the frame rate of 2D scanning on a conventional TPM. For example, with non-resonant galvos running at 1 kHz, we could achieve 1.4 volumes per second (VPS) with a frame size of 512×512 pixels (about 130×130×90μm^3^). The imaging speed can be further improved by limiting the field of view along the slow axis, for instance, 5.6 VPS@ 512×128 pixels (about 130×33×90μm^3^) is achievable. As a comparison, the conventional two-photon microscope with the same scanning speed takes about 100 times longer to acquire the same volumetric image.

### Depth reconstruction method

**Fig 3:**
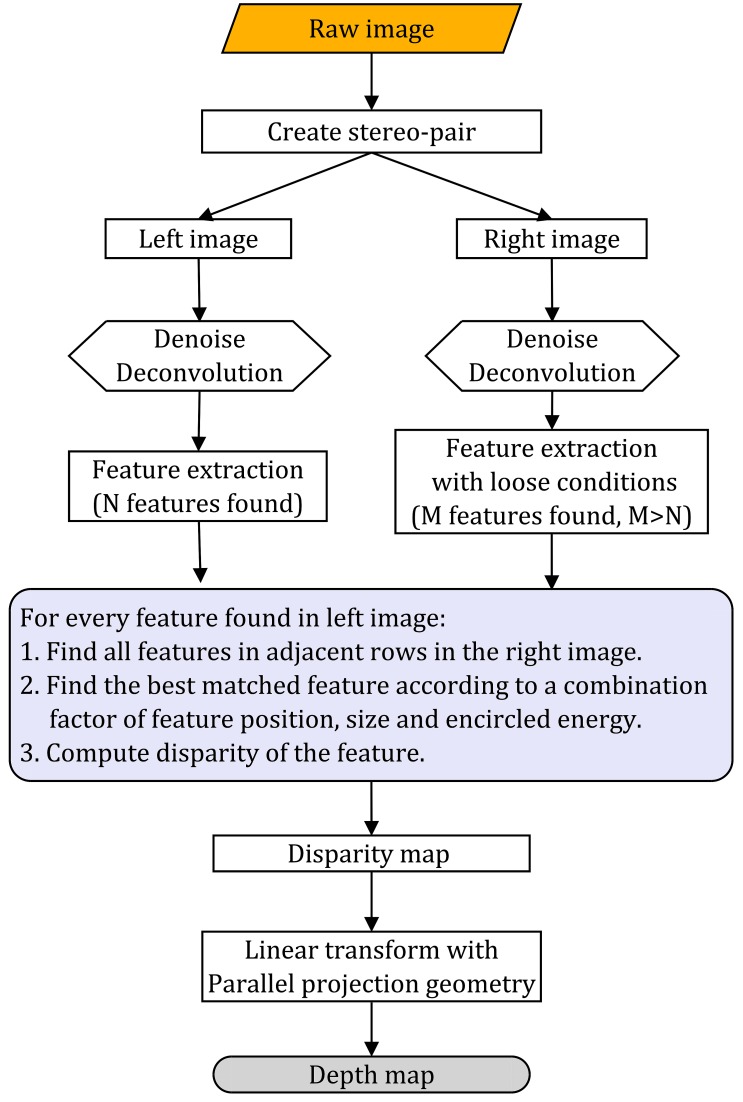
Flowchart of depth-map reconstruction with feature-based method.

In conventional TPM, the depth is directly related to different layers of a z-stack image. However, in TPLSSM, the depth information is encoded in the stereo-pair, inasmuch it is necessary to recover the depth for quantitatively 3D imaging. Although the specimen is not fully sampled along z-direction in TPLSSM as in conventional TPM or tomographic method, the depth information can still be recovered in some cases. If the fluorophore distribution is sparsely dispersed in 3D and presents as recognizable features in the EDF images, the depth information can be reconstructed by the feature-based correspondence algorithms. Fig 3 shows the flowchart of feature extraction and depth map computation for fluorescent beads, where the circular Hough transform [25] is used to find circular objects in the stereo-pair captured by TPLSSM. The stereo-pairs were first processed with a low-pass filter in spatial frequency domain and then deconvolved with Lucy-Richardson algorithm [26]. The correspondence algorithm aims to find the best matched circular object according to the following condition:

Δ=min(ΔE+Δy−S) (1)

where Δ*E* is the normalized energy (summation of pixel values covered by testing object) difference, Δ*y* is the normalized vertical position difference and *S* is the metric of the found circles. To avoid a failed match, a relative loose threshold is used to detect more features in the right image. Algorithms for a computer to recognize features are in general challenging to develop, which owns their own field and is constantly progressing. The algorithm described above is one of simplest ones that are suitable for identifying objects that have simple geometric shapes and sizes. For stereo-pairs containing complex structures, such as pollen grain in our experiment, area-based correspondence algorithms [27] should be used and results may vary greatly between cases; a further investigation needs to be performed and is beyond the gaol of this paper.

## Results

**Fig 4:**
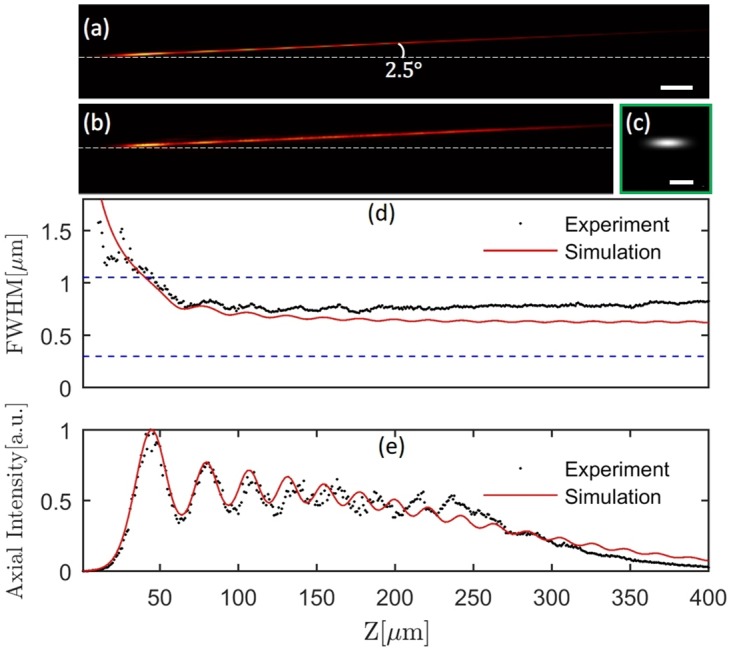
Simulated and measured two-photon excitation PSF2p of Bessel beams with small tilted angle (≈2.5°). (a) Numerical simulation. Scale bar 20μm. (b) Measured PSF2p. (c) PSF2p with Gaussian beam. Scale bar 1μm. (d). Lateral FWHM of PSF2p. (e) Axial intensity distribution along beam direction at different z position.

For precise characterization of TPLSSM, the whole excitation pathway was simulated from collimated Gaussian beam to focal field with real-size optical elements. The simulation included a high NA objective model based on the Rayleigh-Sommerfeld diffraction integral so that a focal volume of about 100μm × 100μm × 500μm could be computed to cover the tilted Bessel beams at any scanned position. Fig 4a shows the simulation of titled PSF2p (square of intensity point spread function), which is consistent with the measured focal field in Fig 4b by the method mentioned in [28]. The lateral FWHM of PSF2p along z direction is shown in Fig 4d. The mean FWHM of measured PSF2p (≈770nm) is worse than the simulation result (≈650nm), since we used an approximate surface model of the axicon instead of its realistic surface profile in simulation. The FWHM of a focused Gaussian beam is about 299nm (lower blue dashed line in Fig 4d) with full aperture illumination, and 1050nm (upper blue dashed line in Fig 4d) with partial aperture illumination, where the beam size is equal to the diameter of the ring pattern at BFP. The experimental axial FWHM (≈160μm) is consistent with the simulation result. The intensity oscillation [29] along beam direction (Fig 4e) is caused by the interference of waves from the round tip and conical surface of the axicon. It is possible to suppress the oscillation [30] such that the lateral resolution and the depth of field will be both greatly improved.

**Fig 5:**
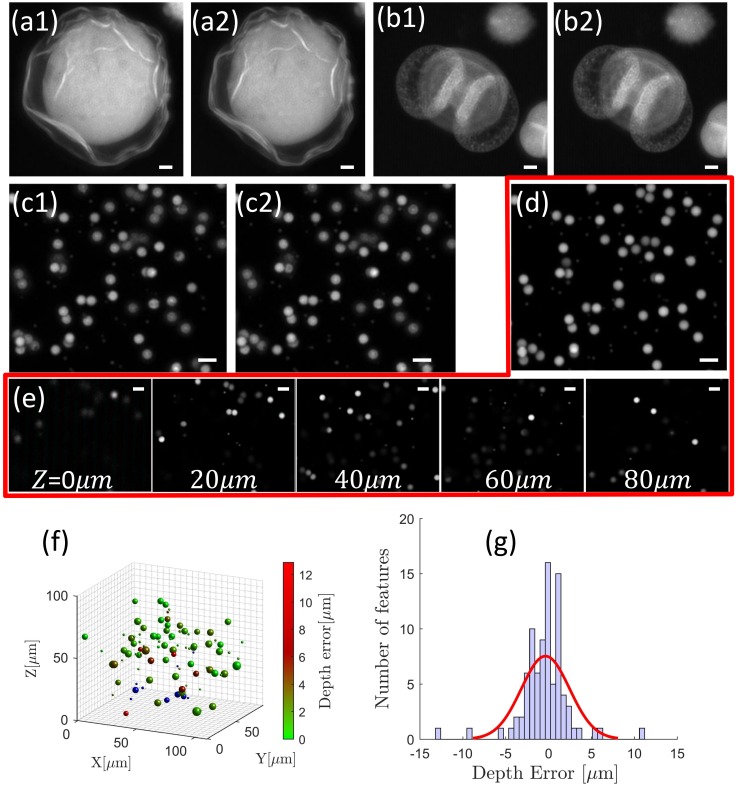
Volumetric imaging and 3D reconstruction. Stereo-pairs of (a-b) pollen grains and (c) fluorescent beads were captured in stereo-mode. (d) Sum projection of the stack along z-axis. (e) 5 selected images from an image stack consisting of 87 slices taken in standard two-photon mode. (f) 3D map of objects with their depth recovered from the stereo-pair in (c). Each object is labeled according to its depth error with a color map that spans from green to red. Blue spheres are unrecognized objects in (e), but are present in (d). (g) Histogram of the depth error with superposed normal distribution (red line). Scale bars in (a)-(e) are all 10μm.

We first demonstrated the imaging performance of the TPLSSM on static thick samples, such as pollen grains and mixed fluorescent beads of 1μm and 6μm in diameter. The high-resolution 3D structures were captured with a single frame, which was then converted to a stereo-pair Fig 5a–5c. These images clearly demonstrate TPLSSM’s penetration ability due to two-photon excitation and “non-diffractive” properties of Bessel beams. S1–S4 Figs show more side-by-side stereo-pairs which can be viewed with 3D shutter glasses. S5 and S6 Figs give anaglyphs merged from stereo-pairs in Fig 5 and more test samples for viewing with red/cyan glasses.

With the feature-based depth reconstruction method, the fluorescent beads shown in Fig 5c can be simply recognized as circular objects with their sizes and locations determined by circular Hough transform. We identified 87 circular objects and obtained their disparities from Fig 5c1 and 5c2. The relative depths of the objects were calculated according to their disparities with triangulation. To determine the accuracy of the recovered depths, we acquired the 3D stack of the same volume with conventional two-photon microscopy for establishing the ground truth values of the depth. We first used the z-projection of the stack (Fig 5d) and the correspondence algorithm to locate the previously identified 87 circular objects, and then determined their depths by searching the maximum intensity along z direction in the 3D stack. The depth map of the objects was used as the ground truth values to evaluate the accuracy of the depths from the stereo-pair. Fig 5f shows all 87 objects with their depth errors labelled with a colour map that spans from green to red. Fig 5g gives the histogram of the depth error. About 70% of the objects are localized axially with depth error less than 2μm, and 93% less than 5μm. Clearly, the depth error cannot be properly described by normal distribution, which is depicted as the superposed red line with its standard deviation σ = 2.82±0.22μm. It is important to note that the localization accuracy [31] is not determined by the axial full-width at half-maximum (FWHM) of Bessel beam, but its lateral FWHM and the correspondence algorithm. The accuracy of the depth information of specimens depends on geometric features of objects and algorithms chosen; thus, the current approach may not suitable for high precision analysis along axial direction.

**Fig 6:**
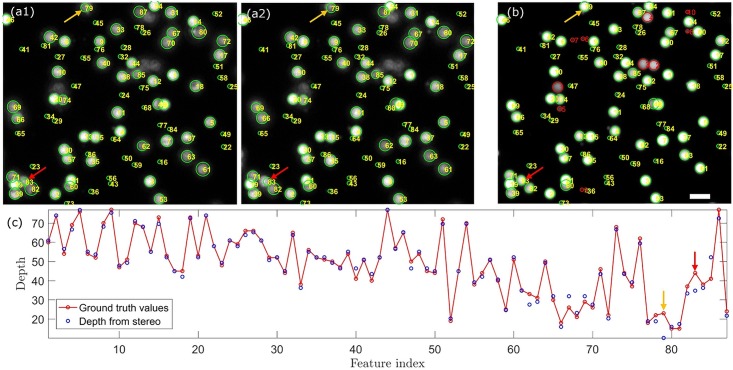
Feature-based correspondence algorithm and the recovered depth. (a) 87 circular objects found in the left image (Fig 5c1). (b) The corresponding circular objects are found in the right image (Fig 5c2). (c) The corresponding circular objects are also found in the z-projection of the stack (Fig 5d) to determine the ground truth depths. (d) Comparing the recovered depths from the stereo-pair and the ground truth values from the image stack.

The detailed processing is shown in Fig 6. The detected circular objects are highlighted by green circles and numbered from 1 to 87. 10 circular objects (highlighted by red circles in Fig 6b, which are depicted as blue spheres in Fig 5f) are not recognized in the stereo-pair (Fig 6a1 and 6a2) due to low contrast. This is likely caused by low fluorophore concentration, or the deterioration of PSF2p at its ends. From Fig 6 we can see that for low contrast or overlapped areas, e.g. feature No.79 (orange arrow) and No.83 (red arrow), the feature detection algorithm may locate the feature deviated from the true position, resulting in erroneous disparities and large depth errors.

**Fig 7:**
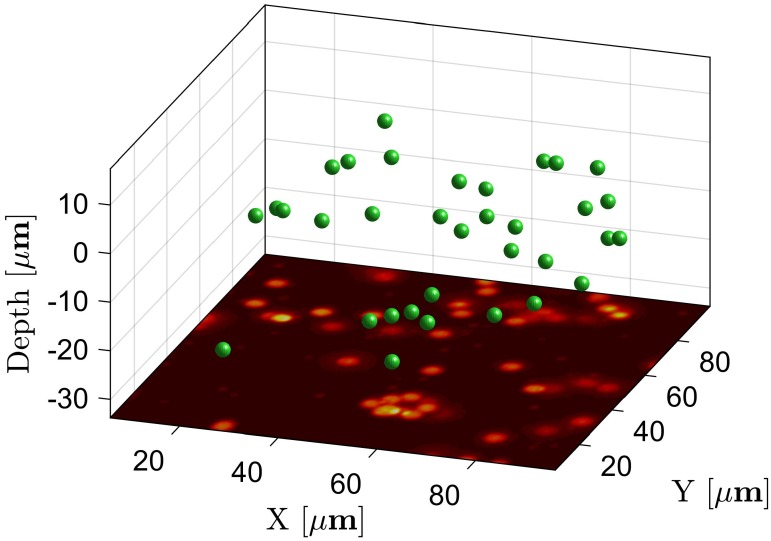
Volumetric imaging of dynamic samples and 3D Reconstruction. The fluorescent beads are moved at an average speed of about 1 μm/s by driving the sample stage. See S1 Movie for the dynamic tracking.

We then demonstrated the performance of the TPLSSM for imaging moving objects by running the sample stage laterally back and forth at various speeds. For 3D tracking of objects, a time sequence of stereo-pairs was recorded and processed by TrackMate [32], a Fiji plug-in for particle tracking. The depth of each tracked object was recovered by applying the correspondence algorithm to each stereo-pair. Fig 7 shows one frame of S1 Movie for 3D dynamic tracking. In fact, the depth information (red/cyan colour in S2 Movie) is maintained at different moving speeds (from 1 to 100 μm/s) in spite of serious distortion due to the relative movements between scanned beams and the sample. The volumetric movement can be directly viewed in real-time by wearing 3D glasses.

## Discussion

The axial resolution dz = dx/sinα is much worse than the lateral resolution dx in TPLSSM, approximate 17μm under the current configuration. In contrast, optical sectioning microscopy, such as spinning-disk confocal scheme [10] or light-sheet scheme [12–14] can achieve axial resolutions of a few micron, which are preferable for high precision quantitative analysis of transparent specimens. However, we have shown experimentally that relative high axial localization accuracy can be achieved provided that the fluorescence distribution can be properly recognized by correspondence algorithms. In fact, the lateral resolution and the correspondence algorithm are two key factors for improving the axial localization accuracy. Fortunately, fluorescent structures of many biological samples present as sparsely distributed features, such as discrete points or filaments, even in the EDF images. Their accurate positions, which may form a 3D pattern or a 3D structure, can provide information about the morphology of the volume of interest. Therefore, the TPLSSM targets those applications in which the volumetric imaging speed is a high priority and limited axial resolution is acceptable. On the contrary, if the structures of interest are dense, the EDF images of a stereo-pair may lose contrast because the projection of fine structures will be heavily overlapped, which inhibits the correspondence algorithm from finding the corresponding features or points and increasing the depth error. To minimize the depth error in this situation, reducing the depth of field may be a strategy, in which chances of overlapping are reduced because of shortening the axial length of the Bessel beam. Engineering the Bessel beam by a spatial light modulator is advantageous for controlling both the lateral resolution and depth of field flexibly, giving the opportunity of improving the depth error.

The imaging speed can be further improved by replacing the fast axis galvo pair with resonant galvo pair, inasmuch tens of Hz volume rate can be achieved with depth information encoded stereoscopically. Although stereo-pairs captured by TPLSSM contain obviously less 3D structural information than 3D image stacks acquired by spinning-disk confocal microscopy or selective plane microscopy, the development of TPLSSM focuses on real-time 3D imaging in scattering media, where volumetric imaging speed is a priority to resolve fast moving objects or dynamic events. The stereoscopic technique described herein is anticipated to be implemented as an add-on imaging mode on a standard two-photon fluorescence microscope to meet different imaging needs in various applications.

## Conclusions

In summary, we have developed two-photon laser scanning stereomicroscopy for imaging 3D fluorescence distribution in real-time with only two frames. By stereoscopically scanning Bessel beams in the excitation pathway, the conventional layout of two optical pathways for microscopic stereo imaging is not a necessity. In addition, the superior penetration ability with two-photon excitation makes it possible to use a high NA objective to capture the dynamic events in turbid medium with a large-area detector. More importantly, for sparse and feature-rich specimens, the depth information can be recovered. With faster scanners, the system can achieve the speed of tens of VPS in scattering medium, and it may provide an efficient way for imaging the neural activity and fast particle velocimetry.

## Supporting Information

S1 Fig Side-by-side stereo-pairs for viewing with 3D shutter glasses. (TIF)

S2 Fig Side-by-side stereo-pairs for viewing with 3D shutter glasses. (TIF)

S3 Fig Side-by-side stereo-pairs for viewing with 3D shutter glasses. (TIF)

S4 Fig Side-by-side stereo-pairs for viewing with 3D shutter glasses. (TIF)

S5 Fig Anaglyphs created by stereo-pairs from TPLSSM. (a-c) Pollen grains. (d) Mixed fluorescent beads of 1μm and 6μm in diameter. Scale bar is 10μm in (a), (b), (c), and (d).
(TIF)

S6 Fig Anaglyphs created by stereo-pairs acquired by TPLSSM. (a-c) Pollen grains. (d) Densely packed fluorescent beads (6μm in diameter) form an inclined surface.
(TIF)

S1 Movie Volumetric imaging and dynamic tracking of dynamic samples. The fluorescent beads were moved at an average speed of about 1 μm/s by driving the sample stage. The left view of the time series images are shown in the bottom plane Z = -32μm, and the right views are not shown. The green spheres represent the detected and tracked fluorescent beads in 3D space.
(MP4)

S2 Movie Stereoscopic imaging of dynamic 3D samples at different moving speeds. The depth information, i.e. the red/cyan channels for left and right views can be appreciated by wearing red/cyan glasses. The stage was paused periodically with different intervals for different average moving speeds. This approach is valid for slow moving speeds, but for fast moving speed (100μm/s), the realistic moving speed is significantly higher than the labeled value, which can be observed by the deformation of the round beads.
(MP4)
