# Supplementary figures and images for: Two-Photon Laser Scanning Stereomicroscopy for Fast Volumetric Imaging
Source: PLoS One. 2016 Dec 20;11(12):e0168885. doi: 10.1371/journal.pone.0168885 (PMC5173245; doi:10.1371/journal.pone.0168885)

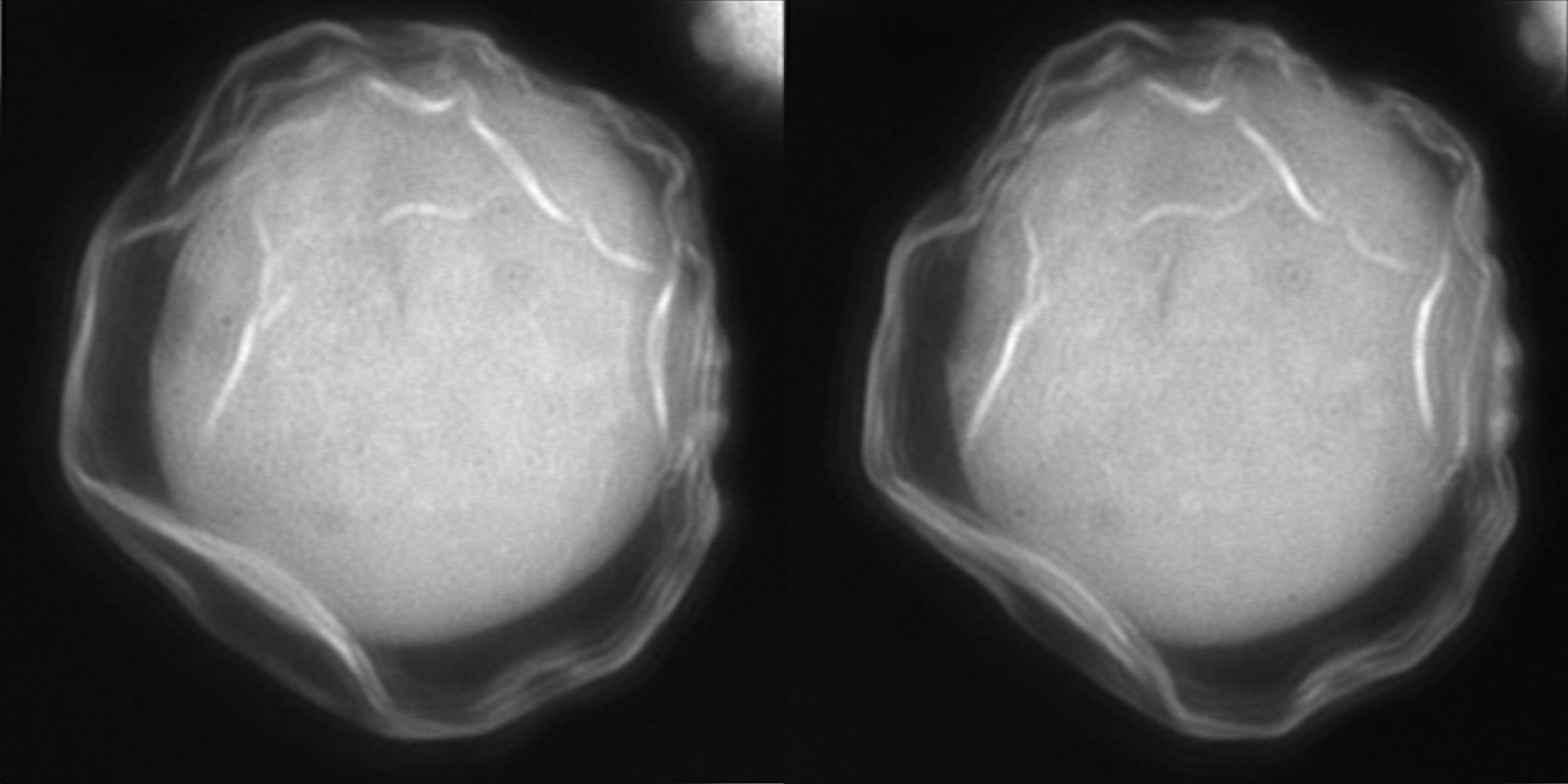

Supplement: S1 Fig — (TIF) [file pone.0168885.s001.tif]

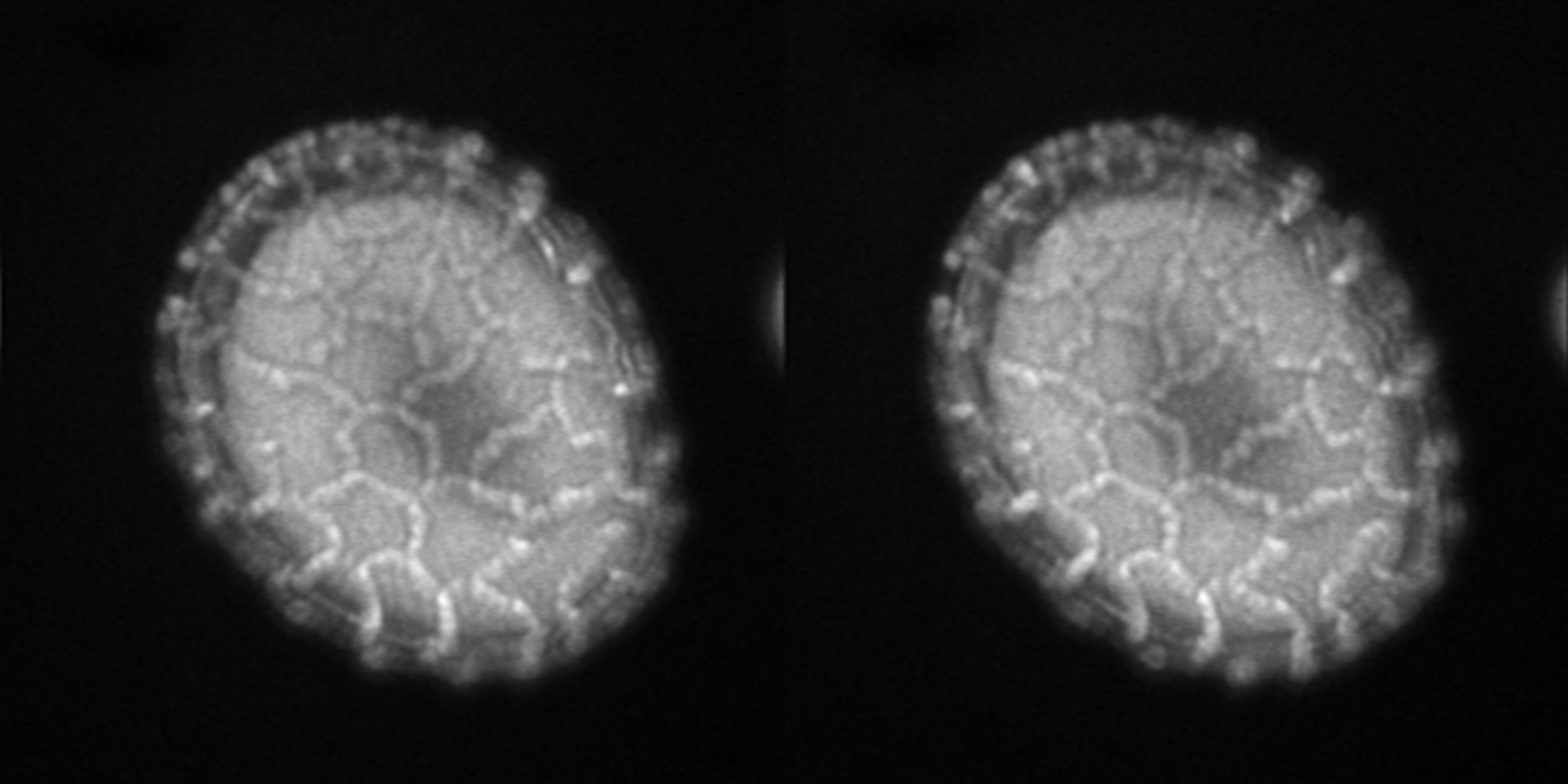

Supplement: S2 Fig — (TIF) [file pone.0168885.s002.tif]

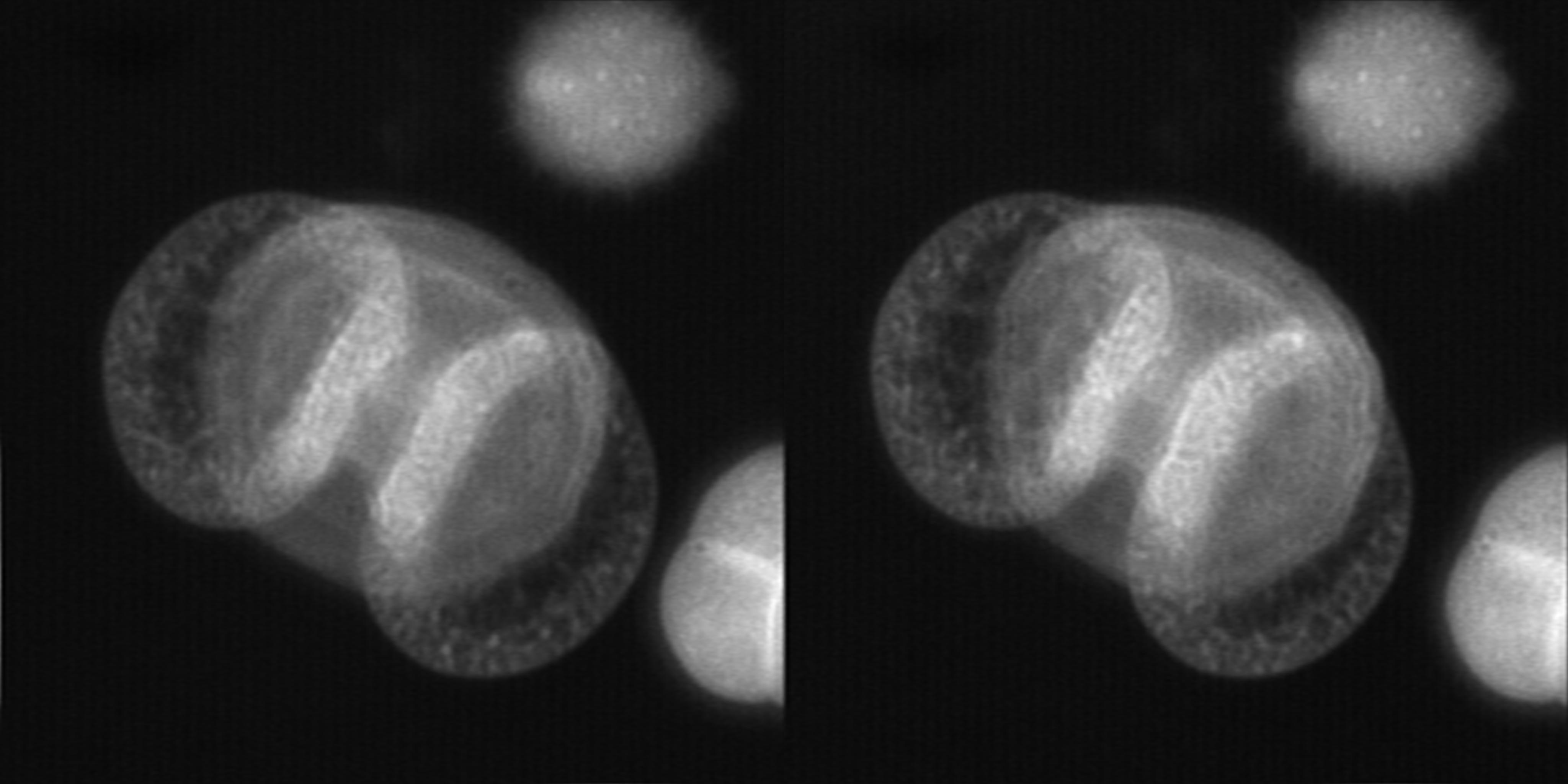

Supplement: S3 Fig — (TIF) [file pone.0168885.s003.tif]

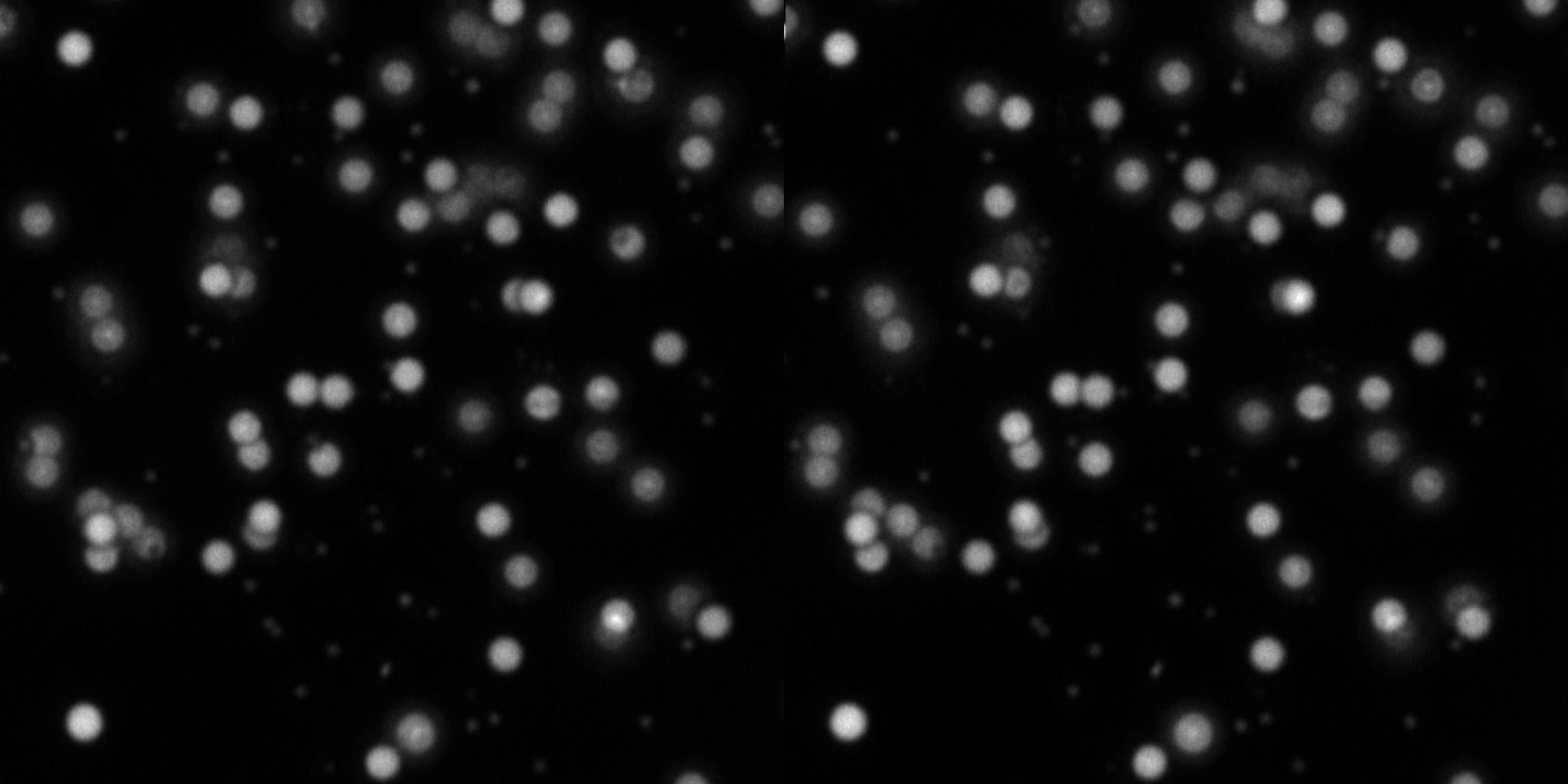

Supplement: S4 Fig — (TIF) [file pone.0168885.s004.tif]

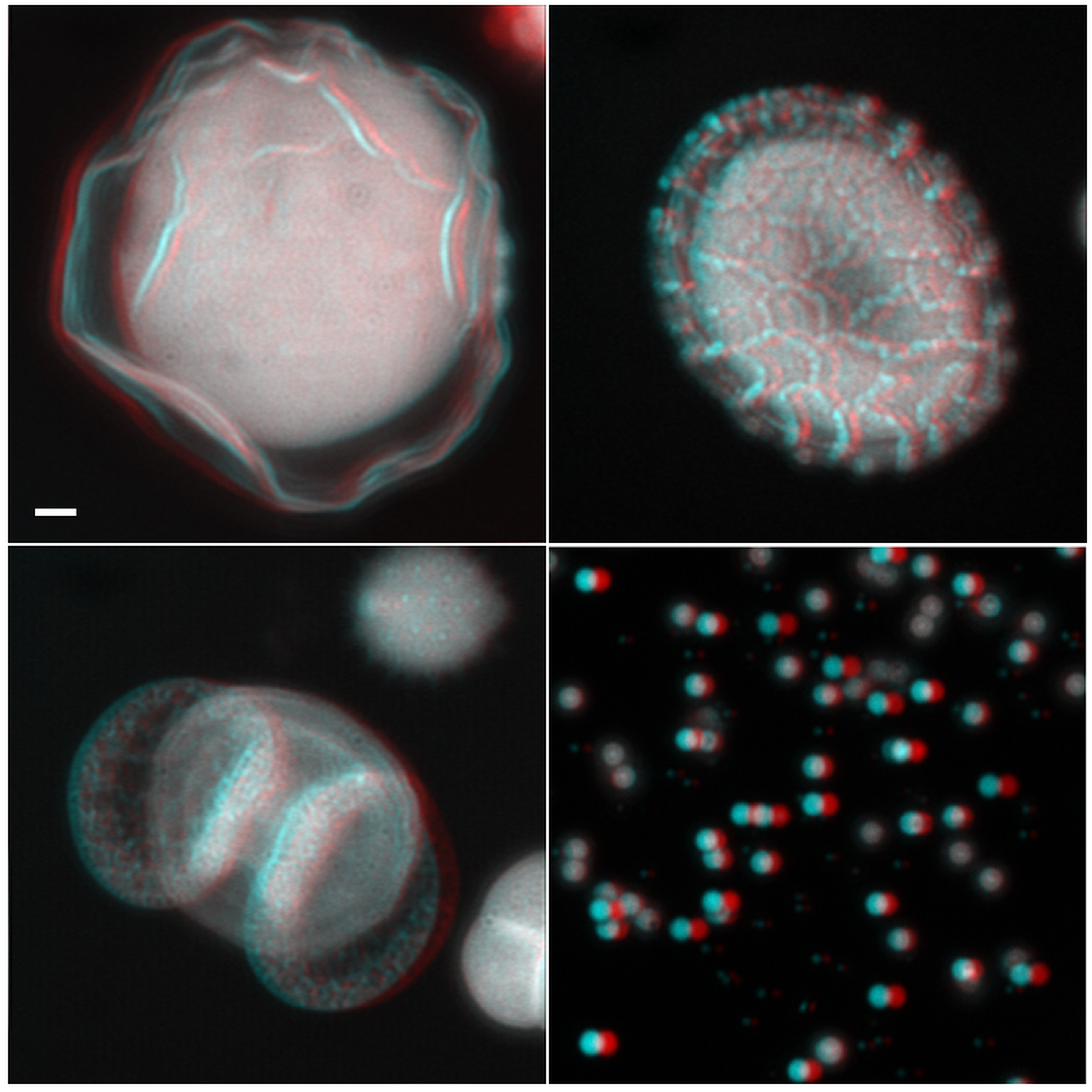

Supplement: S5 Fig — (a-c) Pollen grains. (d) Mixed fluorescent beads of 1μm and 6μm in diameter. Scale bar is 10μm in (a), (b), (c), and (d). (TIF) [file pone.0168885.s005.tif]

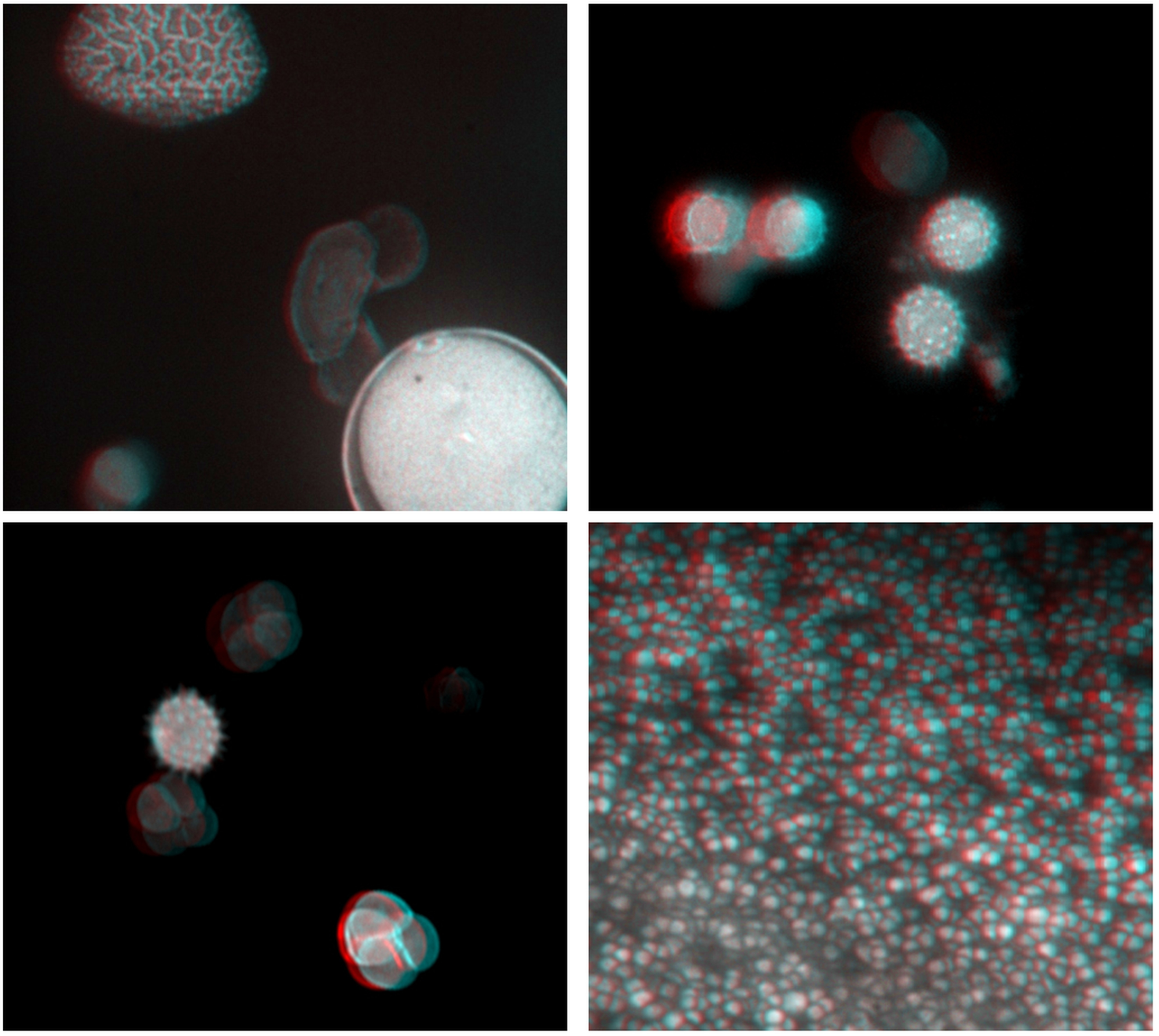

Supplement: S6 Fig — (a-c) Pollen grains. (d) Densely packed fluorescent beads (6μm in diameter) form an inclined surface. (TIF) [file pone.0168885.s006.tif]
